# Supplementary material for: Surface-immobilized cross-linked cationic polyelectrolyte enables CO2 reduction with metal cation-free acidic electrolyte
Source: Nat Commun. 2023 Sep 13;14:5640. doi: 10.1038/s41467-023-41396-2 (PMC10499993; doi:10.1038/s41467-023-41396-2)
Supplement: Supplementary file 1 — Supplementary Information [file 41467_2023_41396_MOESM1_ESM.pdf]

Supplementary Information

Surface-Immobilized Cross-linked Cationic Polyelectrolyte Enables CO_2 Reduction with Metal Cation-free Acidic Electrolyte

AUTHORS: Hai-Gang Qin, Yun-Fan Du, Yi-Yang Bai, Fu-Zhi Li, Xian Yue, Hao Wang, Jian-Zhao Peng, Jun Gu *

AFFILIATION:

Department of Chemistry, Southern University of Science and Technology, Shenzhen, Guangdong 518055, China.

Corresponding author E-mail: guj6@sustech.edu.cn

SIMULATION SECTION

The one-dimensional stationary GMPNP simulation was done with COMSOL Multiphysics version 6.0. The General Form PDE module within COMSOL was used to build the size dependent Nernst Planck equation and the Poisson equation. Geometry of the simulation domain, partial differential equations, boundary conditions, mesh and solver for the finite element analysis are specified below. All parameters and coefficients used in the simulation are listed in Table S2.

Geometry

The geometry of simulation is shown in Figure S20a. The geometry is one-dimensional and consists of two domains. Domain 1 is $x \in [0, L_\mathrm{p}]$, corresponding to the polymer layer. Domain 2 is $x \in (L_\mathrm{p}, L_\mathrm{tot}]$, corresponding to the diffusion layer out of the polymer layer. The thickness of the layer of c-PDDA on Ag MDE was around 1 μm based on the amount of polymer loaded onto the Ag MDE. Therefore, L_p was fixed at 1 μm in most simulations unless otherwise specified. Considering that radius of the Ag MDE (r_0) is 12.5 μm, the effective thickness of the diffusion layer on this Ag MDE is $\frac{\pi}{4} r_0$,[1] namely 9.8 μm. Therefore, L_tot was fixed at 9.8 μm in all simulations.

Size dependent Nernst Planck equations

Figure S20b shows the governing equations and boundary conditions used in the simulations. The size dependent Nernst Planck equation was given by:

$$\frac{\partial C_i}{\partial t} = -\nabla \cdot \boldsymbol{J}_i + R_i \qquad \text{(S1)}$$

where the ∇ operator stands for d/dx, C_i is the concentration of solution specie i (i = H^+, OTf^-, and OH^-), $\boldsymbol{J}_i$ is the flux of specie i and R_i is the production rate of specie i in homogeneous reactions. $\boldsymbol{J}_i$ was given by:

$$\boldsymbol{J}_i = -D_i C_i \nabla(\ln(\gamma_i C_i)) - \frac{D_i C_i z_i F}{RT}\nabla\varphi \tag{S2}$$

In this equation, D_i, C_i and z_i are the diffusion coefficient, concentration and charge of species i, respectively. φ is the potential, F is the Faradaic constant, R is the gas constant, T is the temperature (298 K). γ_i is the activity coefficient. The diffusion coefficients in the polymer layer (Domain 1) were set to 1/10 of the values in water (Domain 2) for each species, which is a commonly used assumption.[2] γ_i in Equation S2 is given by:[3]

$$\gamma_i = \frac{1}{1-V_\mathrm{p}-N_A\sum_{i=1}^{N} a_i^3 C_i} \tag{S3}$$

In this equation, N_A is the Avogadro's number (6.023×10^{23} mol^{-1}). a_i is the effective solvated diameter of the species i. V_p is the volume fraction occupied by the polymer. V_p was set to 0 in the region out of the polymer layer (Domain 2). We observed that the volume of a layer of c-PDDA on a glass slide swelled to 2.8 times of the initial volume of this polymer layer when it was immersed in water (see "*Measurements of charge density of c-PDDA*" in the Methods Section in the main text for the procedure of the measurement). Therefore, V_p was set to 1/2.8 = 0.36 in the polymer layer with ρ_p = +300 C·cm^{-3} (Domain 1). Then, Equation S2 is rewritten as:

$$\boldsymbol{J}_i = -D_i \nabla C_i - \frac{D_i C_i z_i F}{RT}\nabla\varphi - D_i C_i \frac{N_A \sum a_i^3 \nabla C_i}{1-V_\mathrm{p}-N_A\sum a_i^3 C_i} \tag{S4}$$

The polymer with more fixed charge is expected to accommodate more aqueous solution. Therefore, for polymer layers with ρ_p = 0 and ±100 C·cm^{-3}, the volume fraction of aqueous solution ($1 - V_\mathrm{p}$) should be lower than that of polymer layer with ρ_p = +300 C·cm^{-3}. In our simulation, V_p was set to 0.68 for polymer layers with ρ_p = 0 and ±100 C·cm^{-3}. Thus, the volume fraction of aqueous solution in the polymer layer decreases to half of that in the polymer layer with ρ_p = +300 C·cm^{-3}.

The reversible reaction between H^+ and OH^- was considered in the simulation:

$$\mathrm{H_2O} \underset{k_\mathrm{w2}}{\overset{k_\mathrm{w1}}{\rightleftharpoons}} \mathrm{H^+} + \mathrm{OH^-} \tag{S5}$$

where k_w1 is the rate constant of water dissociation, k_w2 is the rate constant of neutralization reaction. Thus, the R_i in Equation S1 is given by:

$R_{OTf-} = 0$ (S6)

$R_{H+} = R_{OH-} = k_{w1} - k_{w2}C_{H+}C_{OH-}$ (S7)

Boundary conditions of the size dependent Nernst Planck equations

OHP is defined as the origin of x axis. The left boundary condition (x = 0) of size dependent Nernst Planck equation is the flux J_i, which is built in COMSOL using the Flux/Source boundary. For OTf^- and OH^-, J_i is given by:

$$J_{ClO4-,x=0} = J_{OH-,x=0} = 0 \quad (S8)$$

For specie H^+, J_i is given by:

$$J_{H+,x=0} = \frac{j_{H+}}{F} \quad (S9)$$

where j_{H+} is the current density of H^+ reduction, The reduction current is defined as negative current. We assume $H^+ + e^- \rightarrow H^*$ (Volmer step) is the rate determining step of H^+ reduction, and this step is irreversible. Thus, the current density can be described as:

$$j_{H^+} = -Fk^0 C_{H^+,x=0} \exp[-\frac{\beta F}{RT}(\varphi_M - \varphi^{0\prime} - \varphi_{x=0})] \quad (S10)$$

In this equation, k^0 is the standard rate constant and φ^{0}' is the formal potential of the rate determining step. β is the transfer coefficient. The term '$-\varphi_{x=0}$' is introduced for the Frumkin correction. φ^{0}' is defined as:[1]

$$\varphi^{0\prime} = \varphi^0 + \frac{RT}{F}\ln\gamma_{H^+} \quad (S11)$$

In this equation, φ^0 is the standard reduction potential of the rate determining step. γ_{H+} is the activity coefficient of H^+ according to Equation S3. We assume k^0, β and φ^0 are constants. Then, Equation S10 can be rewritten as:

$$j_{H^+} = -A\gamma_{H^+}^{\beta} C_{H^+,x=0} \exp[-\frac{\beta F}{RT}(\varphi_M - \varphi_{x=0})] \quad (S12)$$

In this equation, A is a constant expressed as:

$$A = Fk^0 \exp[\frac{\beta F}{RT}\varphi^0] \quad \text{(S13)}$$

The values of A and β were taken from our previous work on the GMPNP simulation of Ag rotating disk electrode.[4]

The right boundary (x = L_{tot}) condition is the concentration of species i in bulk solution, which is built in COMSOL using the Dirichlet Boundary Condition. $C_{i,x=L\mathrm{tot}}$ is given by:

$$C_{\mathrm{H+},x=L\mathrm{tot}} = 0.01\ \mathrm{M} \quad \text{(S14)}$$

$$C_{\mathrm{OH-},x=L\mathrm{tot}} = 10^{-12}\ \mathrm{M} \quad \text{(S15)}$$

$$C_{\mathrm{OTf-},x=L\mathrm{tot}} = 0.01\ \mathrm{M} \quad \text{(S16)}$$

Poisson equation

The Poisson equation is utilized to model the electric field generated from the charged specie, which is built in COMSOL using the General Form PDE module. In the polymer layer (Domain 1), the Poisson equation is given by:

$$\nabla^2\varphi = -\frac{F\sum(z_iC_i)+\rho_\mathrm{p}}{\varepsilon_0\varepsilon_r} \quad \text{(S17)}$$

Out of polymer layer (Domain 2), the Poisson equation is given by:

$$\nabla^2\varphi = -\frac{F\sum(z_iC_i)}{\varepsilon_0\varepsilon_r} \quad \text{(S18)}$$

In above equations, ρ_p is the charge density carried by the polymer layer, ε_0 is the permittivity of vacuum and ε_r is the relative permittivity. The relative permittivity of water was used in both domains. The effect of the polymer on the relative permittivity was not considered in our simulation.

Boundary condition of Poisson equation

The left boundary (x = 0) condition of Poisson equation is the surface excess charge

density of the cathode, which is built in COMSOL using the Flux/Source boundary. The surface charge density of the cathode σ is given by:

$$\sigma = -\varepsilon_0 \varepsilon_r \frac{\varphi_{x=0} - \varphi_M}{d} \quad \text{(S19)}$$

In this equation, $\varphi_{x=0}$ is the potential at OHP, φ_M is the potential at electrode, d is the thickness of Stern layer, which was set to 0.4 nm.

The right boundary (x = L_{tot}) is the potential of bulk solution, which is built in COMSOL using the Dirichlet Boundary Condition. The $\varphi_{x=L\text{tot}}$ is given by:

$$\varphi_{x=L\text{tot}} = 0 \text{ V} \quad \text{(S20)}$$

All potential values used in the simulation is on PZC scale. The PZC of polycrystalline Ag was reported to be -0.55 V vs SHE in acidic solution without specifically adsorbed anions.[5] The simulated potential values were converted to SHE scale by subtracting 0.55 V.

Mesh and solver

The distribution type of mesh is set as predefined, the number of elements is 100000, the element ratio is 0.9999, and the exponential growth rate in reverse direction is selected. The coupled equations were solved with a commercial solver (COMSOL 6.0) utilizing a MUMPS solver with a non-linear automatic Newton method. The solution was converged when a relative tolerance of 0.001 was reached. Further increasing the relative tolerance resulted in a difference in the results of less than 1%.

Supplementary Note 1: Effects of alkali cations and cationic polymer layer on the mass transport of H^+.

Our previous study indicates alkali cations in acidic electrolyte can substantially suppress the migration rate of H^+, but the diffusion of H^+ cannot be significantly inhibited.[4]

In 10 mM HOTf, H^+ and OTf^- are the only ionic species. We consider the diffusion and migration of these two species. Then, their fluxes can be expressed as:

$$J_{x,\mathrm{H}^+} = -D_{\mathrm{H}^+}\frac{\mathrm{d}C_{\mathrm{H}^+}}{\mathrm{d}x} - \frac{D_{\mathrm{H}^+}C_{\mathrm{H}^+}F}{RT}\frac{\mathrm{d}\varphi}{\mathrm{d}x} \quad \text{(S21)}$$

$$J_{x,\mathrm{OTf}^-} = -D_{\mathrm{OTf}^-}\frac{\mathrm{d}C_{\mathrm{OTf}^-}}{\mathrm{d}x} + \frac{D_{\mathrm{OTf}^-}C_{\mathrm{OTf}^-}F}{RT}\frac{\mathrm{d}\varphi}{\mathrm{d}x} \quad \text{(S22)}$$

The first and second terms on the right side of each equation correspond to the diffusion and migration terms, respectively. Considering the electroneutrality, $C_{\mathrm{H}+} = C_{\mathrm{OTf}-} = C$. Since OTf^- is not involved in the electrode reaction, its flux should be zero at steady state. Therefore, we have:

$$\frac{\mathrm{d}C}{\mathrm{d}x} = \frac{CF}{RT}\frac{\mathrm{d}\varphi}{\mathrm{d}x} \quad \text{(S23)}$$

Thus:

$$J_{x,\mathrm{H}^+} = -2D_{\mathrm{H}^+}\frac{\mathrm{d}C}{\mathrm{d}x} = -2\frac{D_{\mathrm{H}^+}CF}{RT}\frac{\mathrm{d}\varphi}{\mathrm{d}x} \quad \text{(S24)}$$

Namely, the migration rate of H^+ equals the diffusion rate of H^+. Therefore, in an electrolyte only containing H^+ and OTf^-, once the migration of H^+ is suppressed by the cationic polymer layer, the diffusion of H^+ is suppressed simultaneously.

This effect can also be understood from Donnan equilibrium:

$$\frac{C_{\mathrm{H}^+,\mathrm{p}}}{C_{\mathrm{H}^+,\mathrm{s}}} = e^{-F(\varphi_\mathrm{p}-\varphi_\mathrm{s})/RT} \quad \text{(S25)}$$

In this equation, $C_{\mathrm{H}+,\mathrm{p}}$ and $C_{\mathrm{H}+,\mathrm{s}}$ represent $C_{\mathrm{H}+}$ in the polymer layer and in the solution, respectively. φ_p and φ_s represent the potential in the polymer layer and in the solution, respectively. Due to the existence of the immobilized positive charge in the positive

layer, φ_p is considerably more positive than φ_s (Figure 5c). Therefore, C_{H+} increases changes abruptly when crossing the interface between the cationic polymer layer and the electrolyte solution. As shown in Figure S23, C_{H+} in the cationic polymer layer is orders of magnitude lower than C_{H+} in the solution, and C_{H+} outside the cationic polymer layer is already quite close to the bulk C_{H+} (0.01 M). Thus, the diffusion rate of H^+ is low due to the low gradient of C_{H+}.

In summary, since the cationic polymer layer suppresses the migration and diffusion of H^+ simultaneously, while the diffusion of H^+ cannot be significantly inhibited by K^+, the plateau current of H^+ reduction on cationic polymer decorated Ag MDE in 10 mM HOTf was lower than that on bare Ag MDE in 10 mM HOTf + 10 mM KOTf.

Supplementary Note 2: GMPNP simulations with varied diffusion coefficients and relative permittivity.

The diffusion coefficient of species i (D_i, i = H^+ and OTf^-) in the polymer layer varies according to the structure of the polymer. We set D_i in the polymer layer to 1/10 of the values in water according to the assumption used by Xu *et al.*[2] To check whether the variation of D_i in the polymer layer ($D_{i,p}$) affects the conclusion of simulation, we tried a series of sets of $D_{i,p}$ values:

$$D_{i,p} = x \cdot D_{i,s} \tag{S26}$$

$D_{i,s}$ is the diffusion coefficient of i in solution shown in Table S2. We tried x = 0.1 and 0.5. Table S3 shows the migration rate of H^+ and the electric field strength in Stern layer (E_{Stern}) at -1.8 V vs SHE obtained from the simulation with different $D_{i,p}/D_{i,s}$ ratios. The variation of $D_{i,p}/D_{i,s}$ ratio did not lead to significant change of E_{Stern}. The migration rate of H^+ increased as the $D_{i,p}/D_{i,s}$ ratio decreased, but the trend that the migration rate of H^+ decreases as ρ_p increases did not change. Therefore, the variation of $D_{i,p}/D_{i,s}$ ratio does not affect the conclusion of the simulation.

The value of local relative permittivity (ε_r) is affected by local concentration of different species and the orientation of molecules. In the simulation results shown in the main text, the value of ε_r in both the polymer layer and solution is set to that of water (80.1). To check whether the variation of ε_r in the polymer layer ($\varepsilon_{r,p}$) affects the conclusion of simulation, we changed $\varepsilon_{r,p}$ to 50.0 and compared the simulation result obtained with $\varepsilon_{r,p}$ of 80.1. Table S4 shows the migration rate of H^+ and E_{Stern} at -1.8 V vs SHE obtained from the simulation with different $\varepsilon_{r,p}$. The decrease of $\varepsilon_{r,p}$ did not lead to significant change of the migration rate of H^+. E_{Stern} increased as $\varepsilon_{r,p}$ decreased, but the trend that E_{Stern} increases as ρ_p increases did not change. Therefore, the variation of $\varepsilon_{r,p}$ does not affect the conclusion of the simulation.

Due to the orientation of water molecules in the Stern layer and the chemisorption on the cathode, ε_r in the Stern layer is typically greatly lower than that in bulk solution. Zhu *et al.* assumed a drop of over 90% in ε_r in the Stern layer.[6] To check whether the

variation of ε_r in the Stern layer affects the conclusion of simulation, we modified the value of ε_r in the Stern layer in Equation S19 to 10% of ε_r of water (8.01) and conducted the GMPNP simulation. Table S5 shows the migration rate of H^+ and E_{Stern} at -1.8 V vs SHE obtained from the simulation with different ε_r in the Stern layer ($\varepsilon_{r,\mathrm{Stern}}$). The decrease of $\varepsilon_{r,\mathrm{Stern}}$ did not lead to significant change of the migration rate of H^+. E_{Stern} increased drastically as $\varepsilon_{r,\mathrm{Stern}}$ decreased, but the difference among the values of E_{Stern} at varied ρ_{p} kept almost unchanged as $\varepsilon_{r,\mathrm{Stern}}$ decreased. Considering that the electric field in Stern layer is the energetic driving force of CO_2 reduction, the decrease of $\varepsilon_{r,\mathrm{Stern}}$ would not lead to the change of the relative rate of CO_2 reduction obtained with different ρ_{p}. In summary, the variation of $\varepsilon_{r,\mathrm{Stern}}$ does not affect the conclusion of the simulation.

In the GMPNP simulations on CO_2 reduction conducted by Bohra et al.[7] and Butt *et al.*,[8] the local value of ε_r is correlated with the local concentration of cations:

$$\varepsilon_r = \varepsilon_0^r \left(\frac{M_{\mathrm{water}} - \sum_i w_i c_i}{M_{\mathrm{water}}}\right) + \varepsilon_r^{\mathrm{min}} \left(\frac{\sum_i w_i c_i}{M_{\mathrm{water}}}\right) \qquad \text{(S27)}$$

In this equation, ε_0^r is the relative permittivity of pure water (80.1), $\varepsilon_r^{\mathrm{min}}$ is the relative permittivity of water under dielectric saturation (6), M_{water} is the molarity of water at 25 °C (55 M) and w_i is the number of water molecules held by the cation i. We also tried to correlate the local ε_r with the local concentration of cation in the GMPNP simulation with the same equation, but we failed to get convergent result.

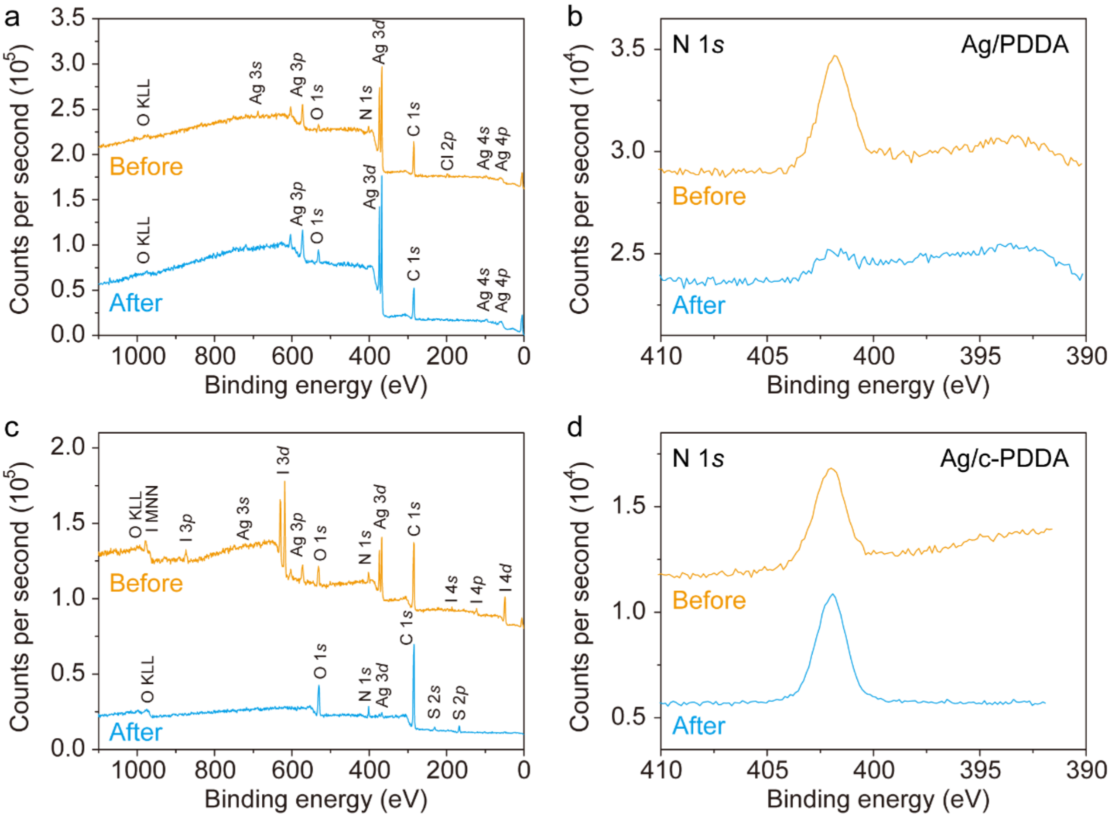

Figure S1. (a, c) Survey XPS and (b,d) N 1*s* XPS spectra of (a, b) PDDA decorated Ag NPs and (c, d) c-PDDA decorated Ag NPs before (orange curves) and after (blue curves) CO_2 electroreduction experiments. I element was detected in the as-prepared c-PDDA decorated Ag NPs, which was introduced from 1,6-diiodohexane. I element existed in the form of I^- ions and were exchanged by SO_4^{2-} ions during electrolysis in 0.1 M H_2SO_4, as indicated by that S element was detected in the c-PDDA decorated Ag NPs after electrolysis. The intensity of Ag peaks of c-PDDA decorated Ag NPs decreased drastically after electrolysis. This does not indicate the leaching of Ag, since ICP-MS measurement showed that Ag was not dissolved in the electrolyte. This observation can be ascribed to that the thickness of c-PDDA layer increased when the catalyst was immersed in water. The thicker layer of polymer inhibited the photoelectrons generated by Ag from being detected.

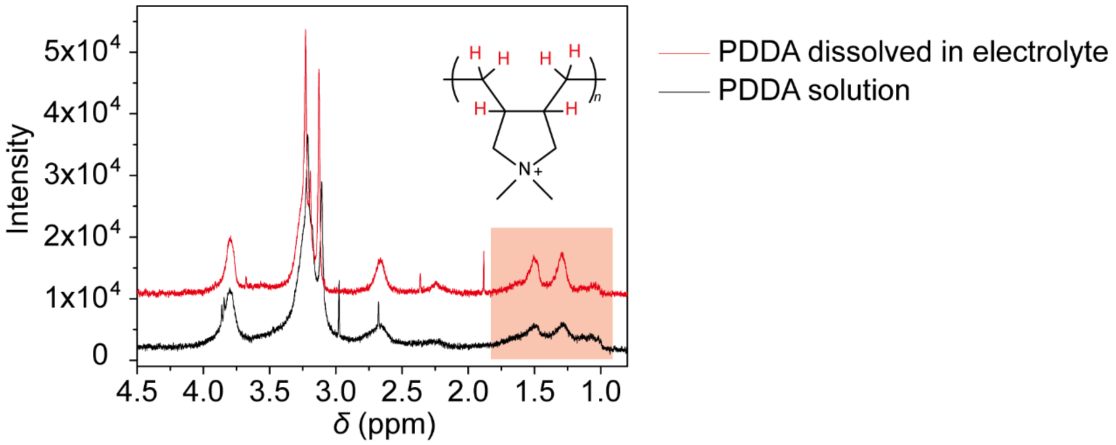

Figure S2. ^{1}H-NMR spectra of PDDA in the electrolyte after CO_2 reduction experiment (red curve) and the standard reference solution of PDDA (black curve). The catalyst was PDDA decorated Ag NPs and the electrolyte was 0.1 M H_2SO_4. After electrolysis, the electrolyte was neutralized by KOH and water was removed by evaporation. The residue was then dissolved by 400 μL of D_2O as the sample for NMR analysis. For the standard reference, PDDA solution containing the same amount of PDDA as that added on the working electrode was distilled to remove the water and then dissolved by 400 μL of D_2O. The signal with the chemical shift between 1.0 and 1.8 ppm is assigned to the CH and CH_2 moieties unbound to nitrogen atom (red H atoms in the inset), which was used to quantify the amount of PDDA dissolved by the electrolyte.

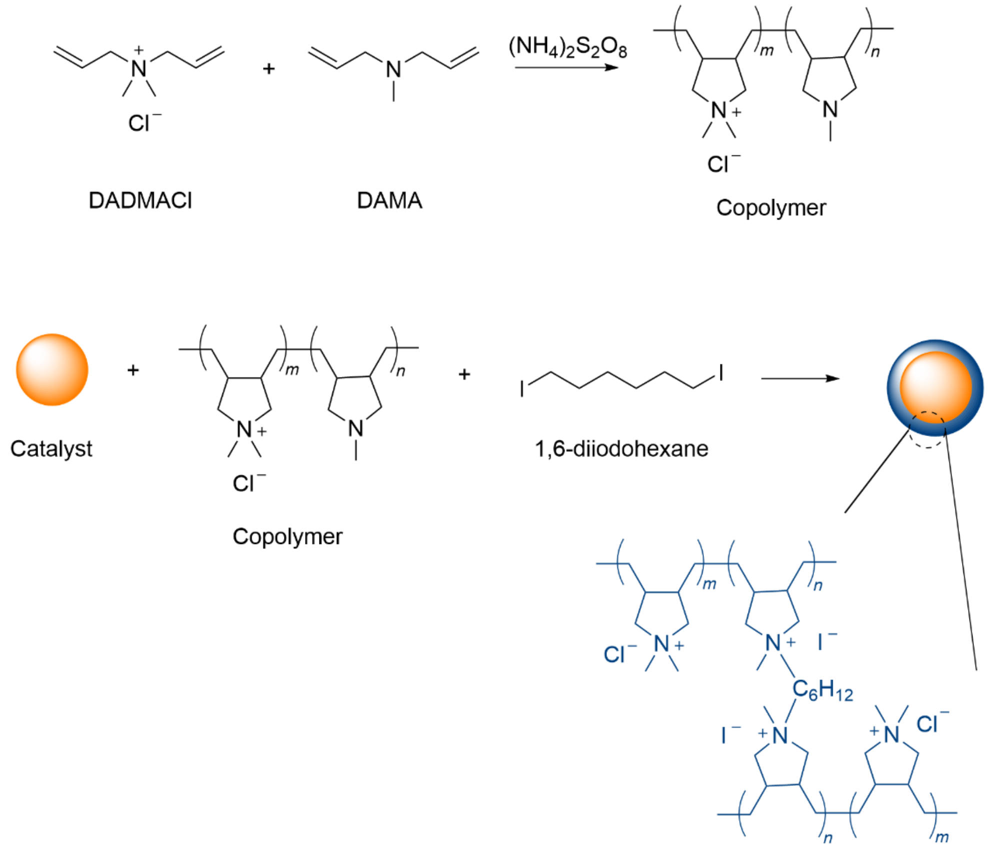

Figure S3. Procedure of the preparation of c-PDDA decorated catalyst.

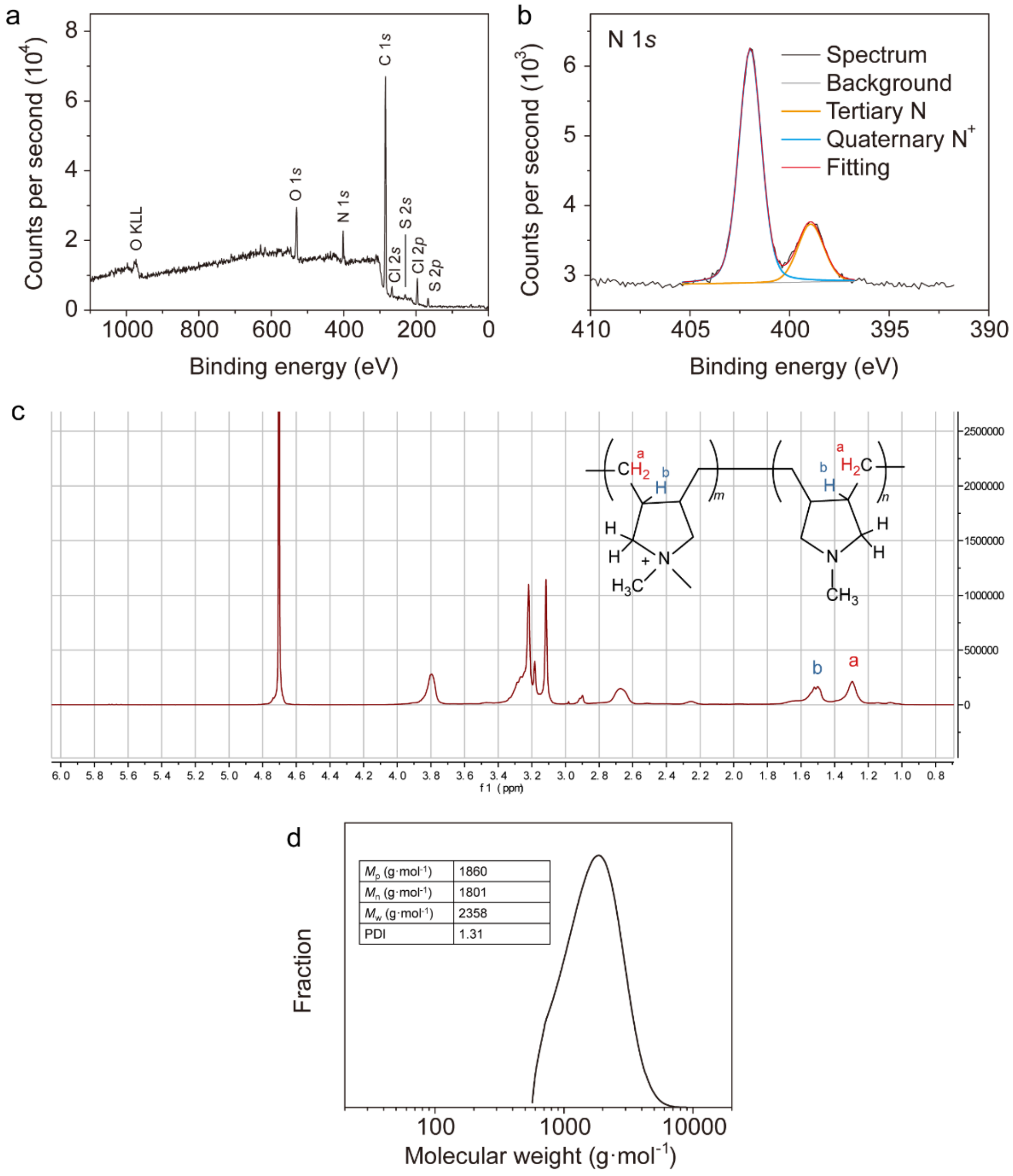

Figure S4. Characterizations of the copolymer of DADMACl and DAMA. (a) Survey XPS spectrum. (b) N 1*s* XPS spectrum. The spectrum is deconvoluted into two peaks corresponding to tertiary amine sites (orange) and quaternary ammonium sites (blue). (c) ^{1}H-NMR spectrum. The broadened peaks indicate the formation of polymer. Peaks with the chemical shift of 5~6 corresponding to CH_2=CH- moieties disappeared, and peaks with the chemical shift of 1.2~1.7 corresponding to CH_2 at the γ site of N (peak a) and CH at the β site of N appeared, confirming the polymerization reaction. (d) The distribution of molecular weight (MW) determined by GPC with water as the mobile phase. M_p, M_n, M_w and PDI represent peak MW, number averaged MW, weight averaged MW and polymer dispersity index, respectively.

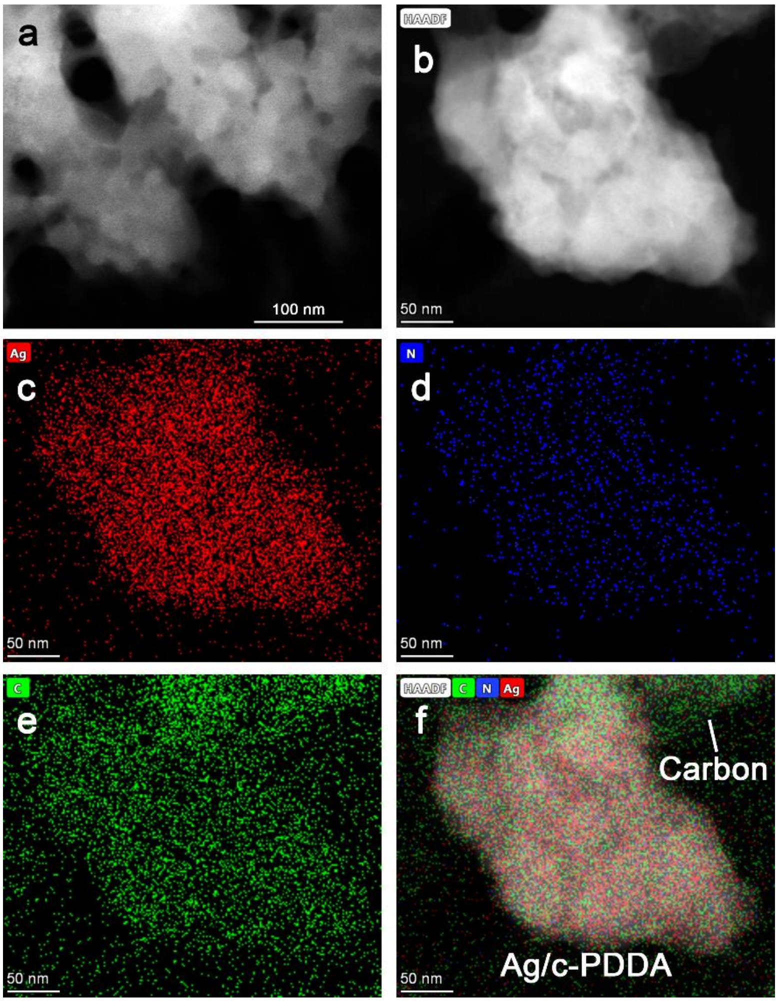

Figure S5. (a, b) HAADF-STEM images of Ag NPs decorated by c-PDDA. (c-f) EDS mapping of the region in panel (b). (c) Ag channel, (d) N channel, (e) C channel and (f) merging of the three channels. The sample was peeled off from a piece of GDE and dispersed in ethanol with sonification. Carbon particles on the GDE were peeled off together with the catalyst. Therefore, Ag element and N element appear in the same region while C element appears in a larger region, as indicated in panel (f).

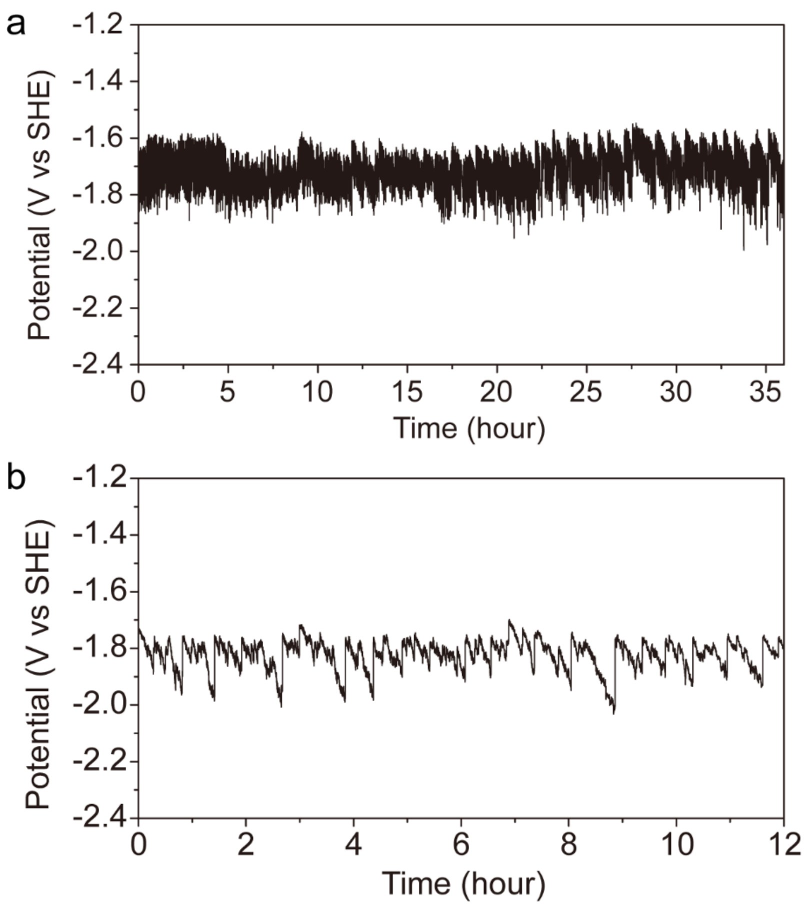

Figure S6. Chronopotentiometry curves of (a) c-PDDA decorated Ag NPs in 0.1 M H_2SO_4 and (b) bare Ag NPs in 0.1 M H_2SO_4 + 0.4 M K_2SO_4. The total current density was -200 mA·cm^{-2}.

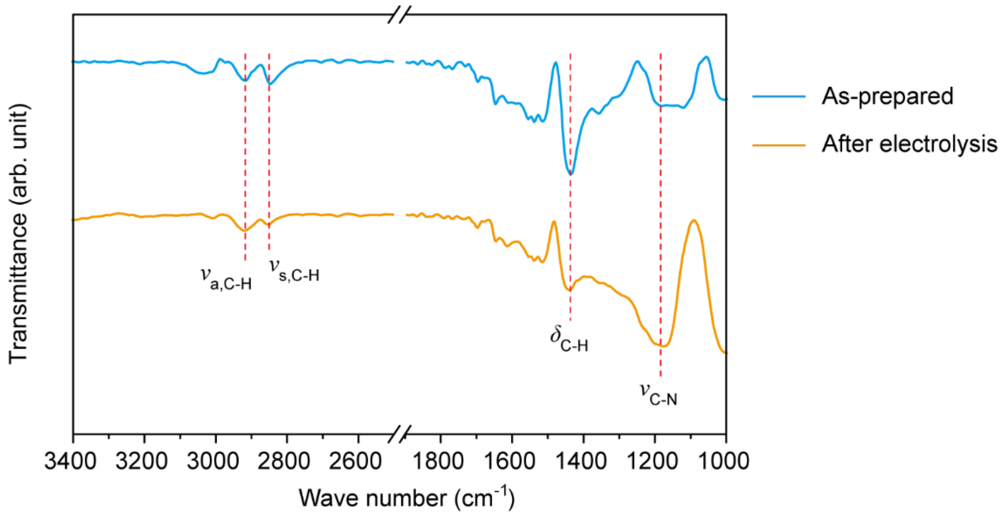

Figure S7. IR spectra of c-PDDA decorated Ag NPs on GDE before and after CO_2 reduction experiment. The stretching vibration of C-H and C-N bonds, and the bending vibration of C-H bonds are assigned.

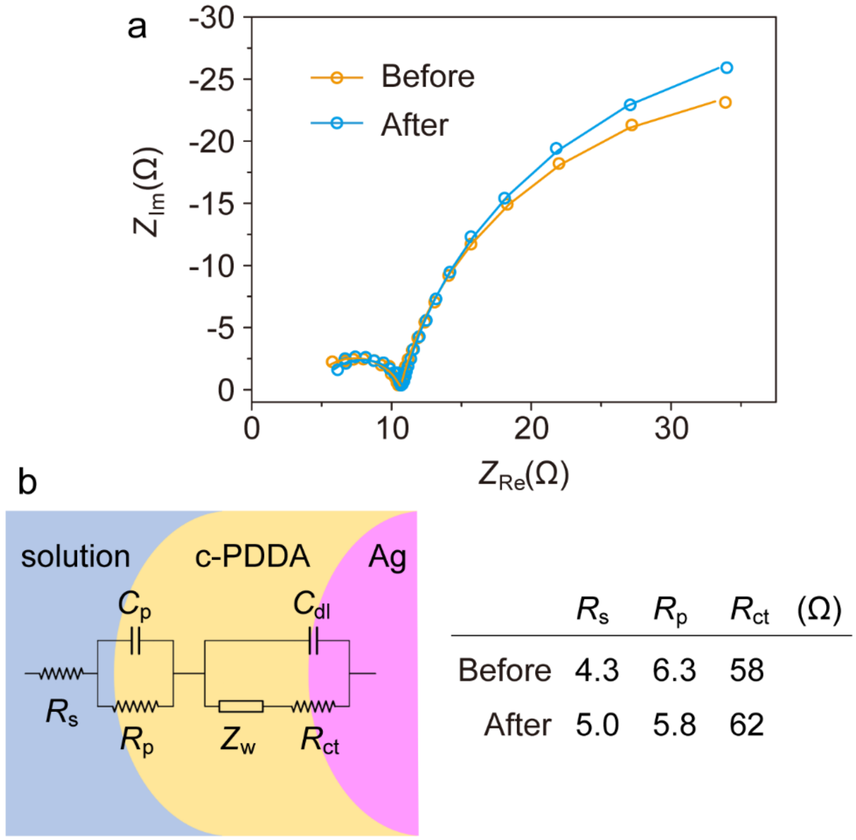

Figure S8. (a) EIS spectra data (circles) and fitting curves (solid lines) and (b) the fitting circuit of c-PDDA decorated Ag NPs on GDE in 0.1 M H_2SO_4 before and after CO_2 reduction experiment. The central potential was -1.0 V vs SHE. The hollow circles and solid lines in panel (a) are experimental data and fitting curves, respectively. In the fitting circuit, R_s, R_p and R_{ct} represent the resistance of solution, polymer layer and charge transfer at the surface of catalyst, respectively. C_p and C_{dl} are the capacitance of polymer layer and the electric double layer, respectively. Z_w is the Warberg impedance. The fitting values of R_s, R_p and R_{ct} are shown.

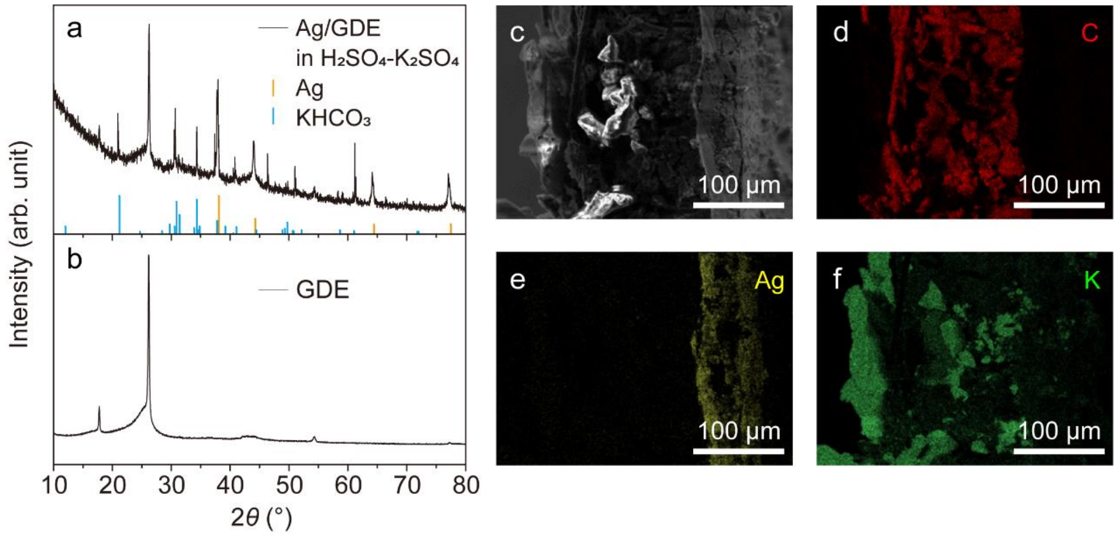

Figure S9. Characterizations of GDE with bare Ag NPs after CO_2 electroreduction with 0.1 M H_2SO_4 + 0.4 M K_2SO_4 as the electrolyte. XRD patterns of (a) Ag/GDE after electrolysis and (b) bare GDE. The orange and blue vertical lines indicate the standard diffraction peaks of face-centered-cubic Ag (JCPDS card no. 04-0783) and monoclinic $KHCO_3$ (JCPDS card no. 12-0292), respectively. (c) SEM image and (d-f) the corresponding EDS mapping images of the cross-section of Ag/GDE after electrolysis. Panels (d)-(f) show the distribution of C, Ag and K elements, respectively.

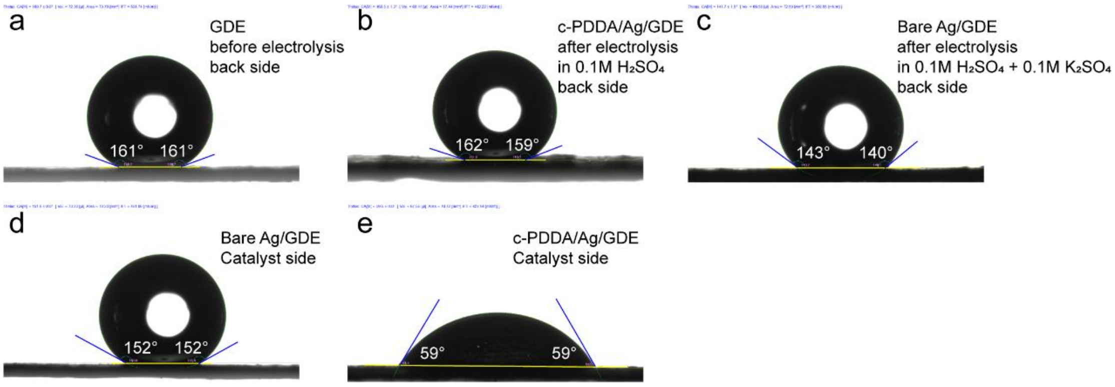

Figure S10. Contact angles of GDEs with bare Ag NPs and c-PDDA decorated Ag NPs. (a) Back side (gas diffusion layer side) of GDE before CO_2 reduction. (b) Back side of c-PDDA/Ag/GDE after CO_2 reduction in 0.1 M H_2SO_4. (c) Back side of bare Ag/GDE after CO_2 reduction in 0.1 M H_2SO_4 + 0.4 M K_2SO_4. (d) Catalyst side of bare Ag/GDE before CO_2 reduction. (e) Catalyst side of c-PDDA/Ag /GDE before CO_2 reduction.

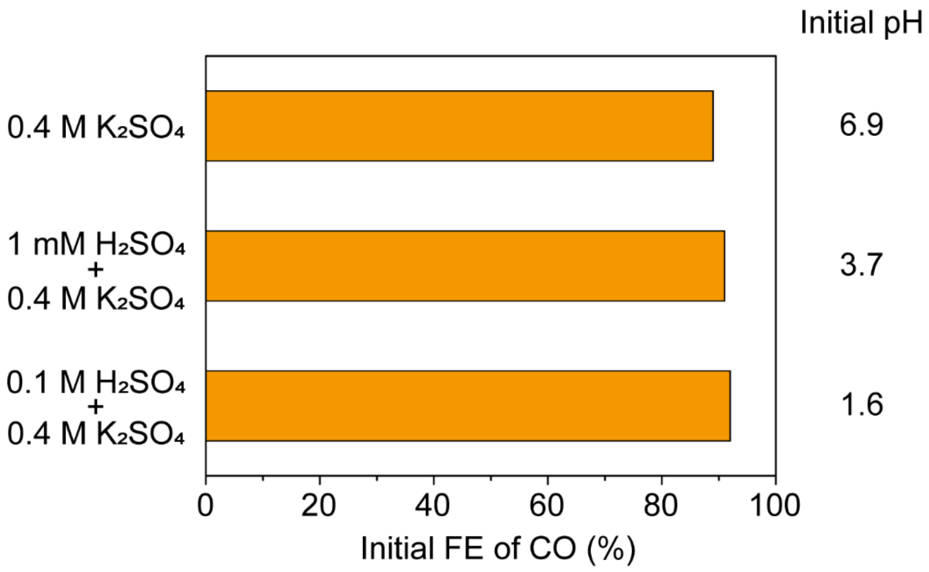

Figure S11. FEs of CO on bare Ag NPs in K^{+}-containing electrolyte with different pH. The electrolyte contained x M H_2SO_4 + 0.4 M K_2SO_4 (x = 0, 0.001, 0.1). The current density was -200 $mA \cdot cm^{-2}$. The FE was measured at 10 minutes of electrolysis.

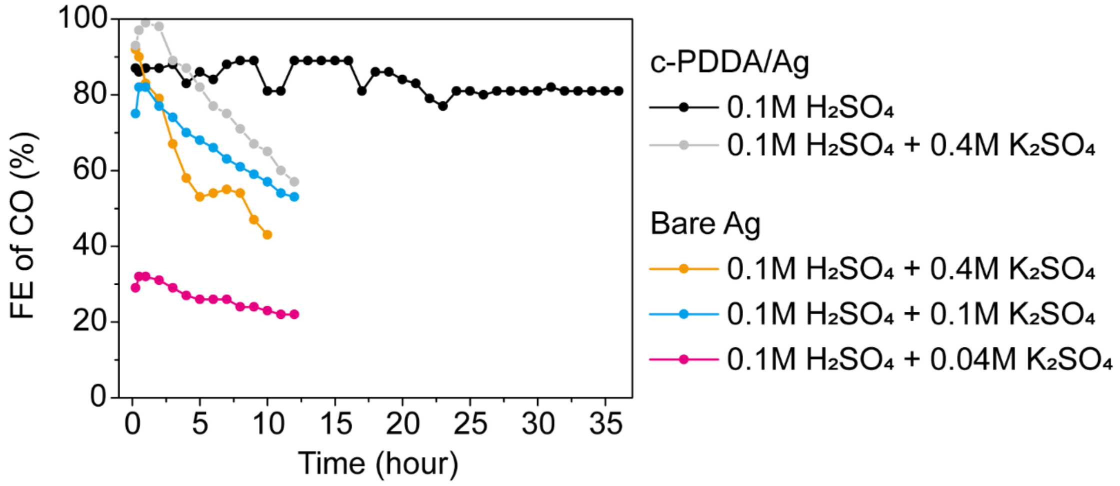

Figure S12. The FE of CO during electrolysis with constant current density of -200 $mA \cdot cm^{-2}$ on bare Ag NPs in 0.1 M H_2SO_4 + x M K_2SO_4 ($x = 0.04 \sim 0.4$) and on c-PDDA decorated Ag NPs in 0.1 M H_2SO_4 or in 0.1 M H_2SO_4 + 0.4 M K_2SO_4.

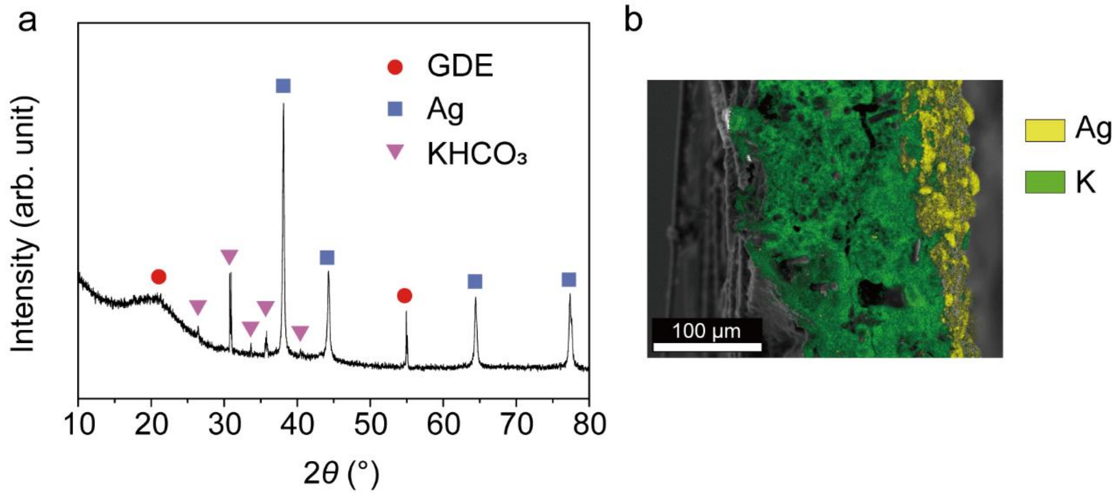

Figure S13. Characterizations of GDE with c-PDDA decorated Ag NPs after CO_2 electroreduction with 0.1 M H_2SO_4 + 0.4 M K_2SO_4 as the electrolyte. (a) XRD patterns. Red circles, blue squares and pink triangles represent the diffraction peaks from GDE, Ag and $KHCO_3$, respectively. (b) SEM images and EDS mapping of the cross-sections of c-PDDA/Ag/GDE after electrolysis. Yellow and green regions represent Ag and K elements, respectively. K element was detected in the gas diffusion layer of the GDE after electrolysis.

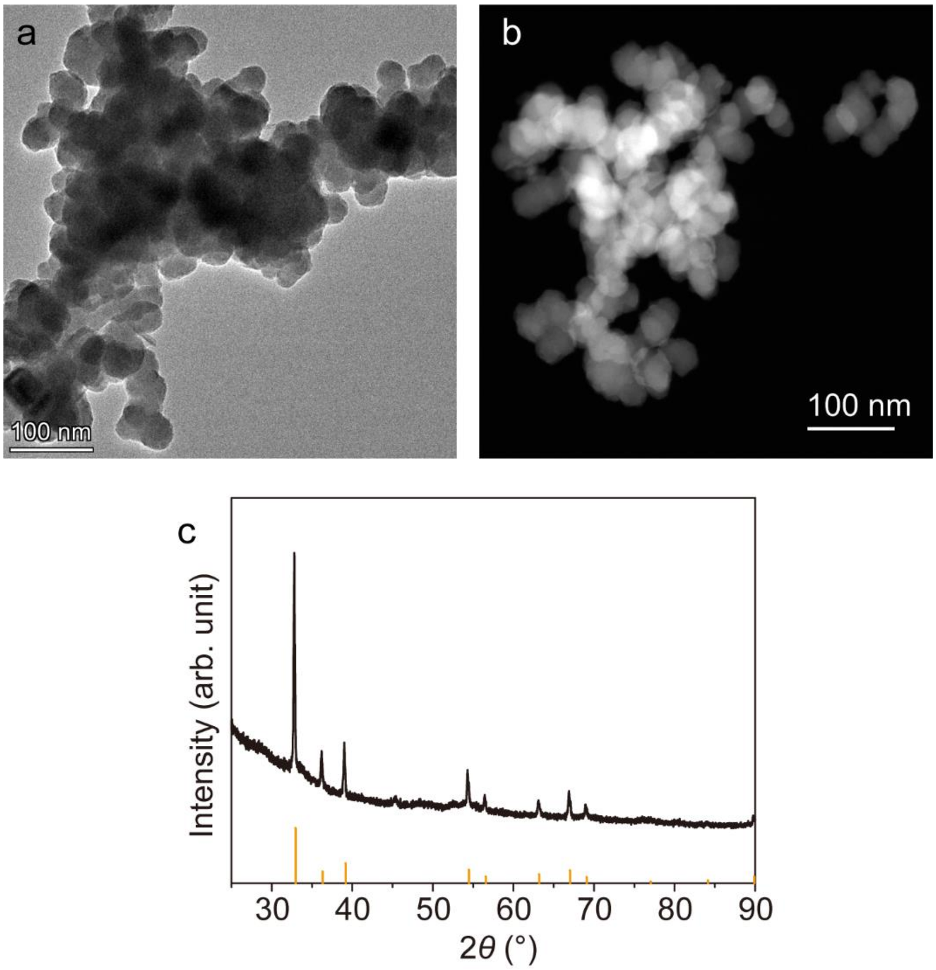

Figure S14. Characterizations of In NPs. (a) TEM image. (b) HAADF-STEM image. (c) XRD pattern. The vertical lines indicate the standard diffraction pattern of tetragonal In (JCPDS No. 05-0642).

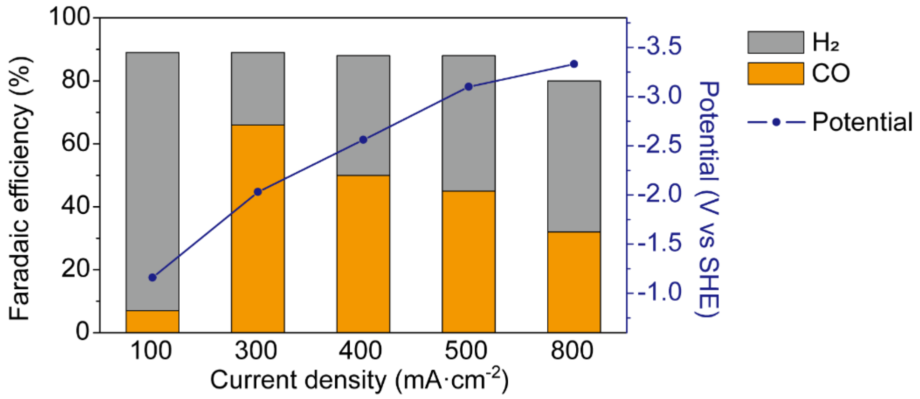

Figure S15. Faradaic efficiency and electrode potential of CO_2 reduction on c-PDDA decorated Ag NPs with 1 M H_2SO_4 as the electrolyte.

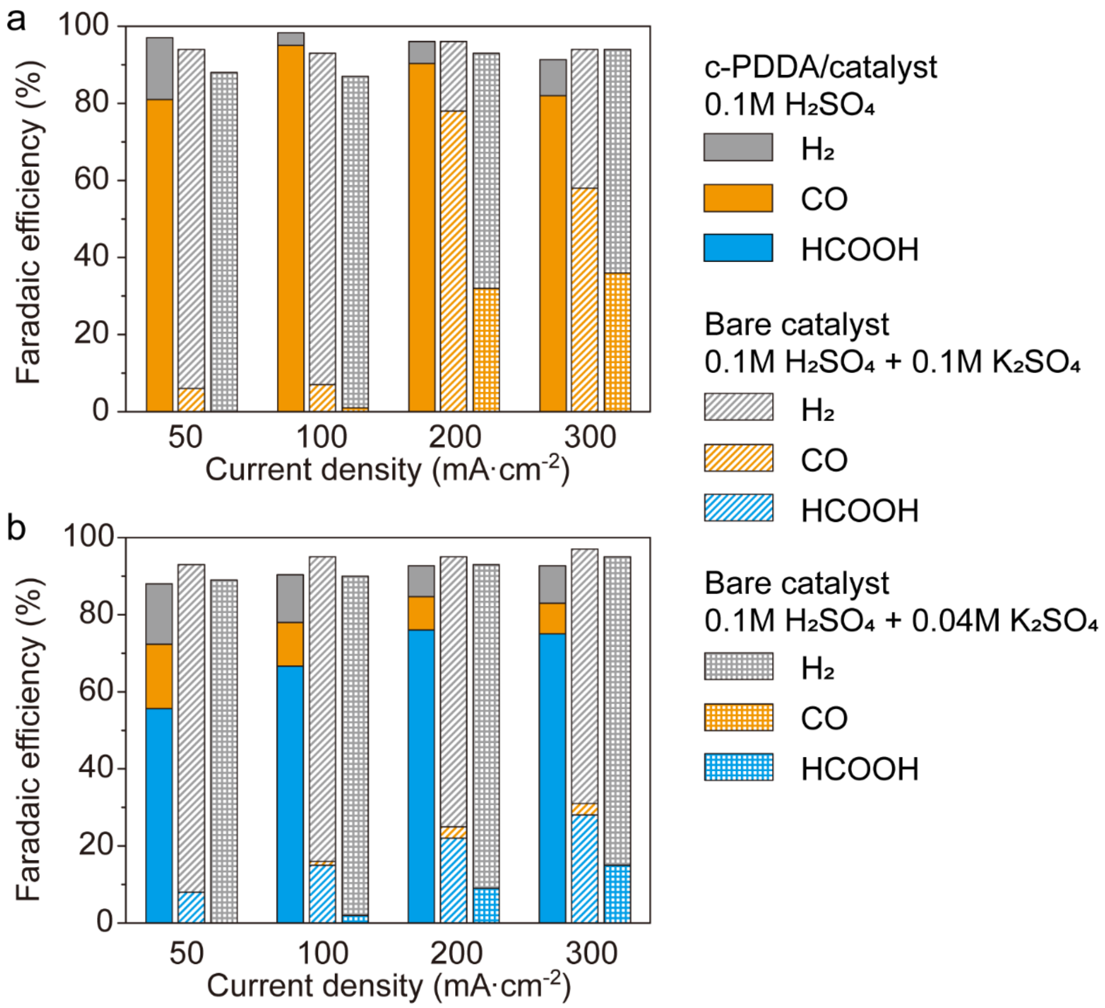

Figure S16. Comparison of FEs of (a) Ag NPs and (b) In NPs at varied current density. c-PDDA decorated catalysts in 0.1 M H_2SO_4 and bare catalysts in 0.1 M H_2SO_4 + 0.1 M K_2SO_4 and 0.1 M H_2SO_4 + 0.04 M K_2SO_4 are compared.

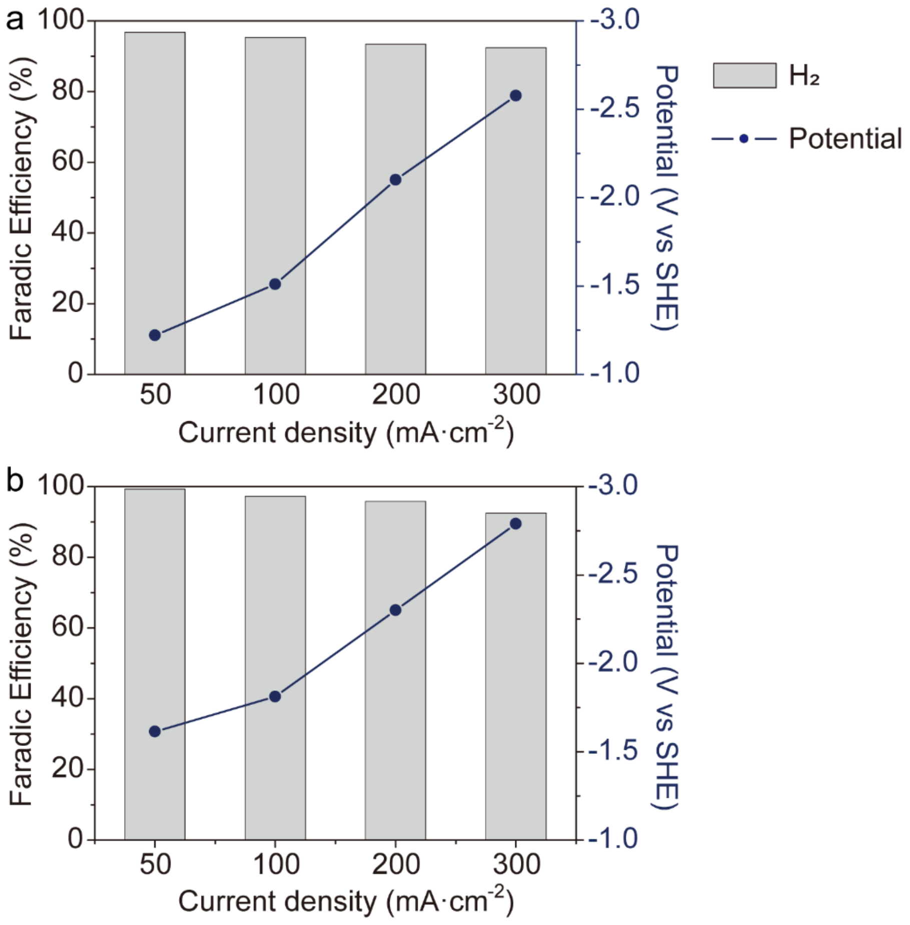

Figure S17. Faradaic efficiency and electrode potential of (a) bare Ag NPs and (b) bare In NPs by feeding CO_2 and with 0.1 M H_2SO_4 as the electrolyte.

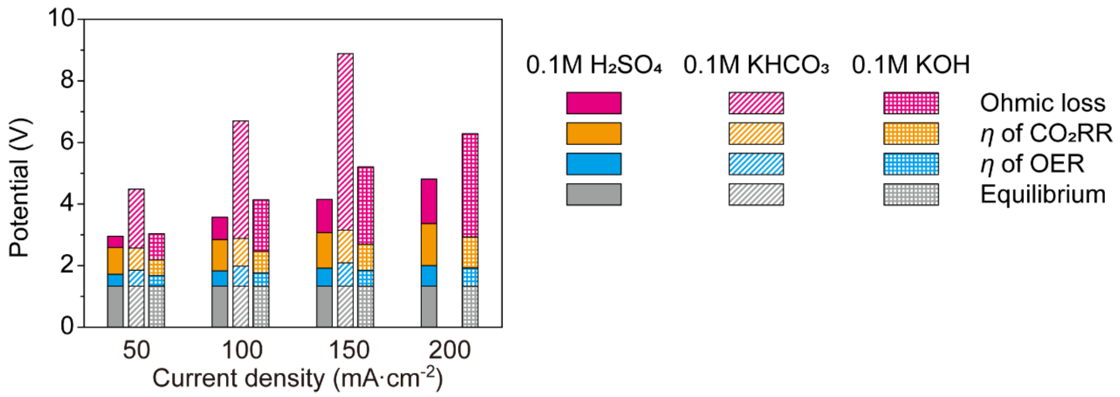

Figure S18. Comparison of the overall cell potential and the potential losses due to electrolyte resistance (Ohmic loss), cathode overpotential (η of CO_2RR) and anode overpotential (η of OER) at varied current densities with different electrolytes. 0.1 M H_2SO_4, 0.1 M $KHCO_3$ and 0.1 M KOH were used as the electrolytes. The cathode was c-PDDA decorated Ag NPs on GDE. The anode used in acidic and near neutral electrolytes was an IrO_2-decorated Ti foil, and the anode used in alkaline electrolyte was an Fe-decorated Ni foam.[9]

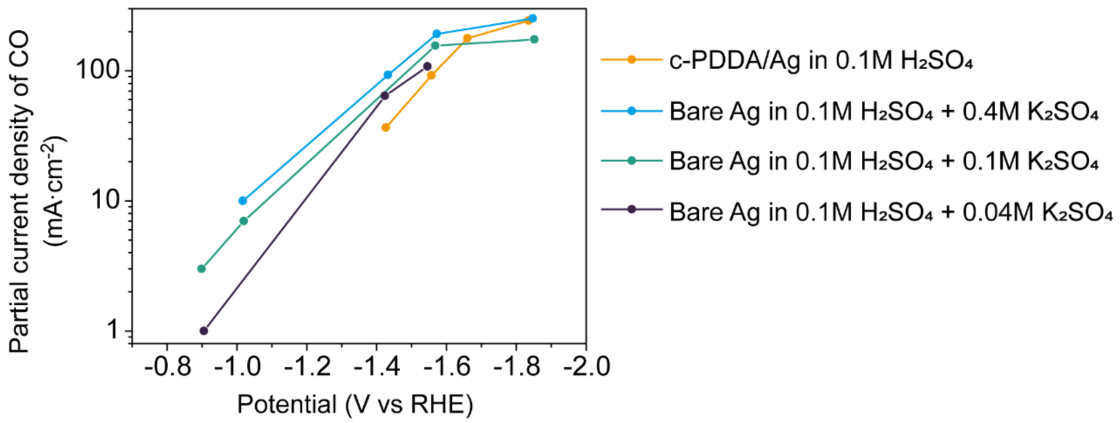

Figure S19. Plots of partial current density of CO dependent on the potential of working electrode for c-PDDA decorated Ag NPs in 0.1 M H_2SO_4 and bare Ag NPs in 0.1 M H_2SO_4 + 0.4 M K_2SO_4, 0.1 M H_2SO_4 + 0.1 M K_2SO_4 and 0.1 M H_2SO_4 + 0.04 M K_2SO_4.

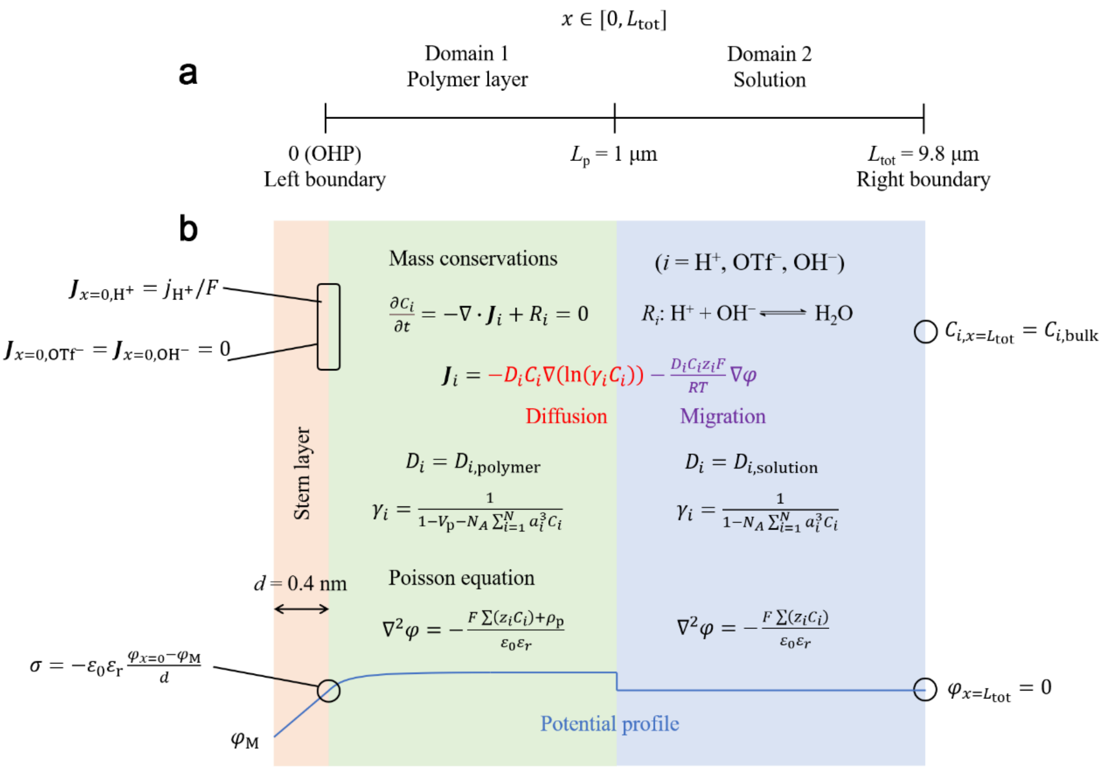

Figure S20. Schematic of the GMPNP simulation. (a) Geometry. (b) Governing equations and boundary conditions.

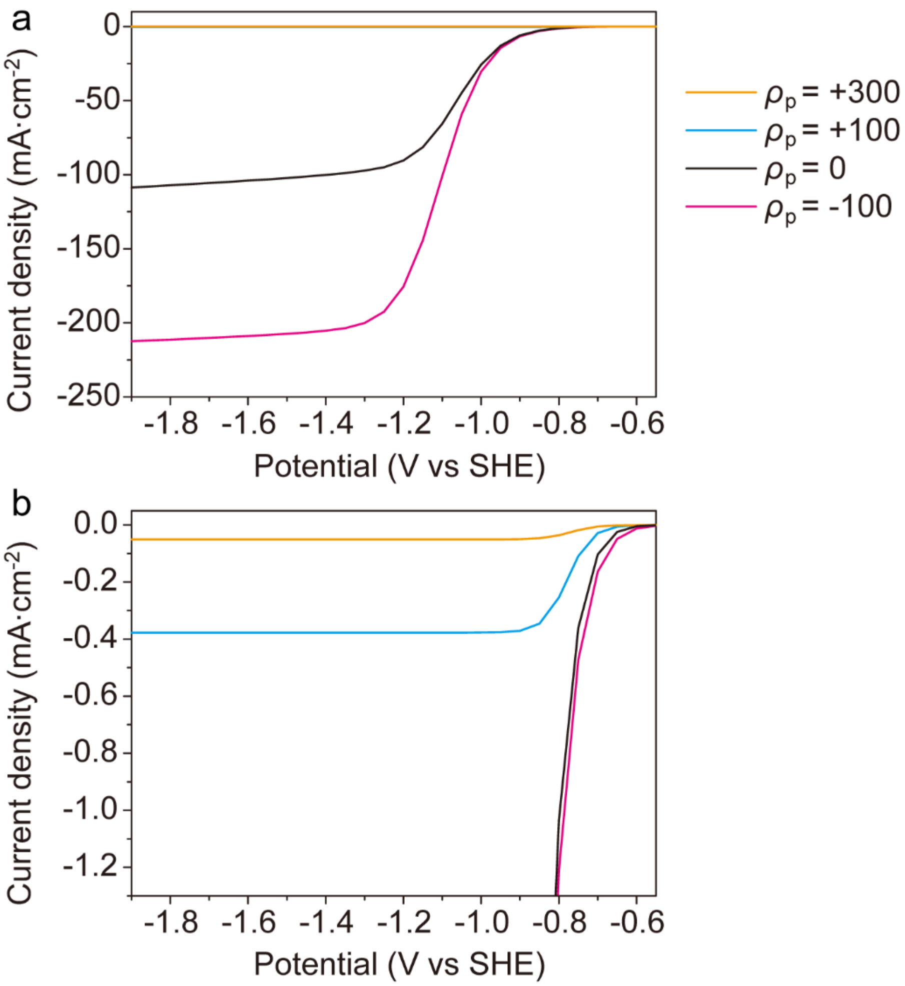

Figure S21. Simulated polarization curves of H^+ reduction of Ag electrode covered by polymer layer with different ρ_p (unit: $C \cdot cm^{-3}$) in 10 mM HOTf. (b) is the magnification of (a).

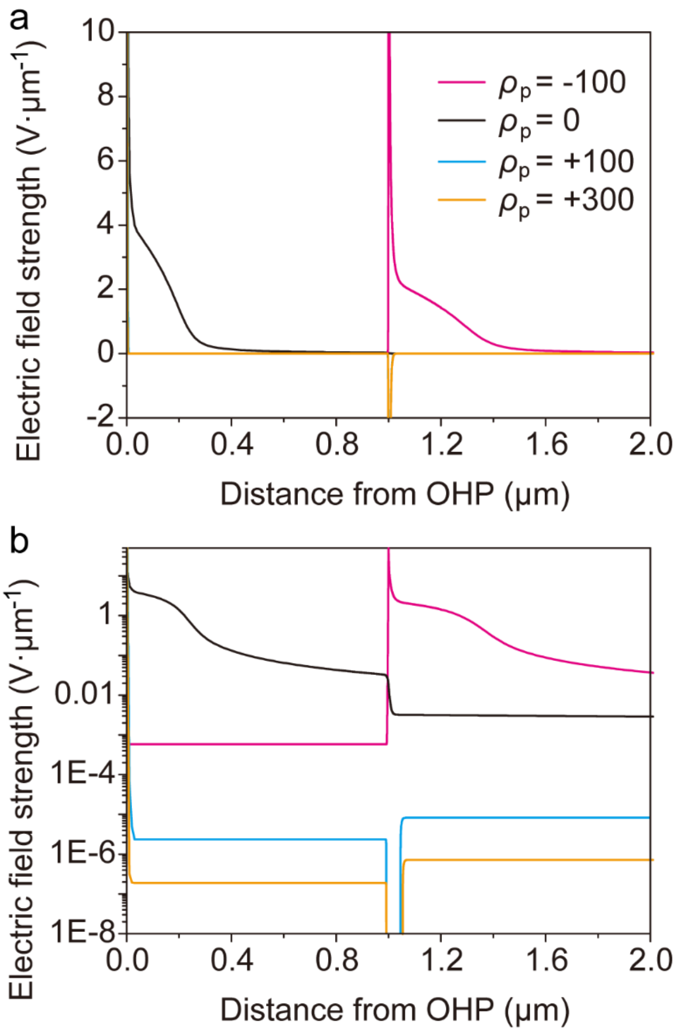

Figure S22. Profiles of electric field strength toward the cathode on Ag electrode covered by polymer layer with different ρ_p (unit: $C\cdot cm^{-3}$) in 10 mM HOTf. (a) Linear scale. (b) Logarithmic scale.

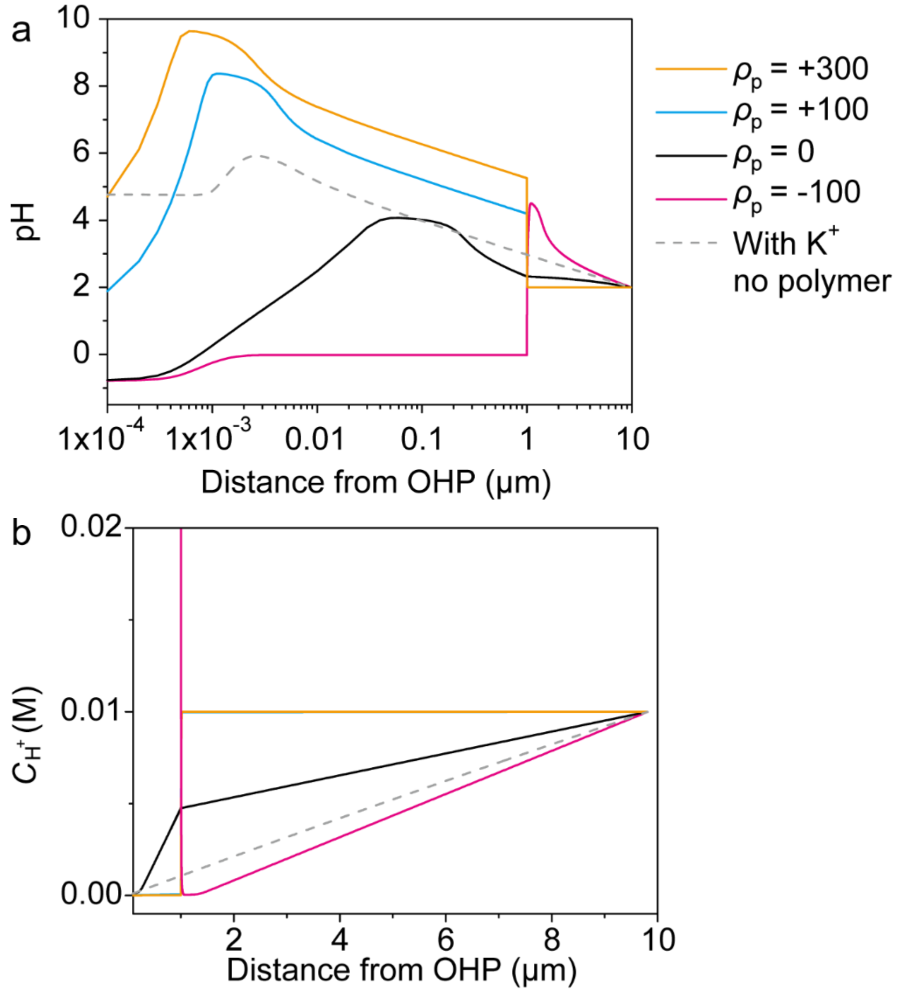

Figure S23. Simulated (a) pH and (b) C_{H+} profiles at -1.8 V vs SHE. Solid curves: Ag electrode covered by polymer layer with different ρ_p (unit: C·cm^{-3}) in 10 mM HOTf. Dashed grey curve: bare Ag electrode in 10 mM HOTf + 40 mM KOTf.

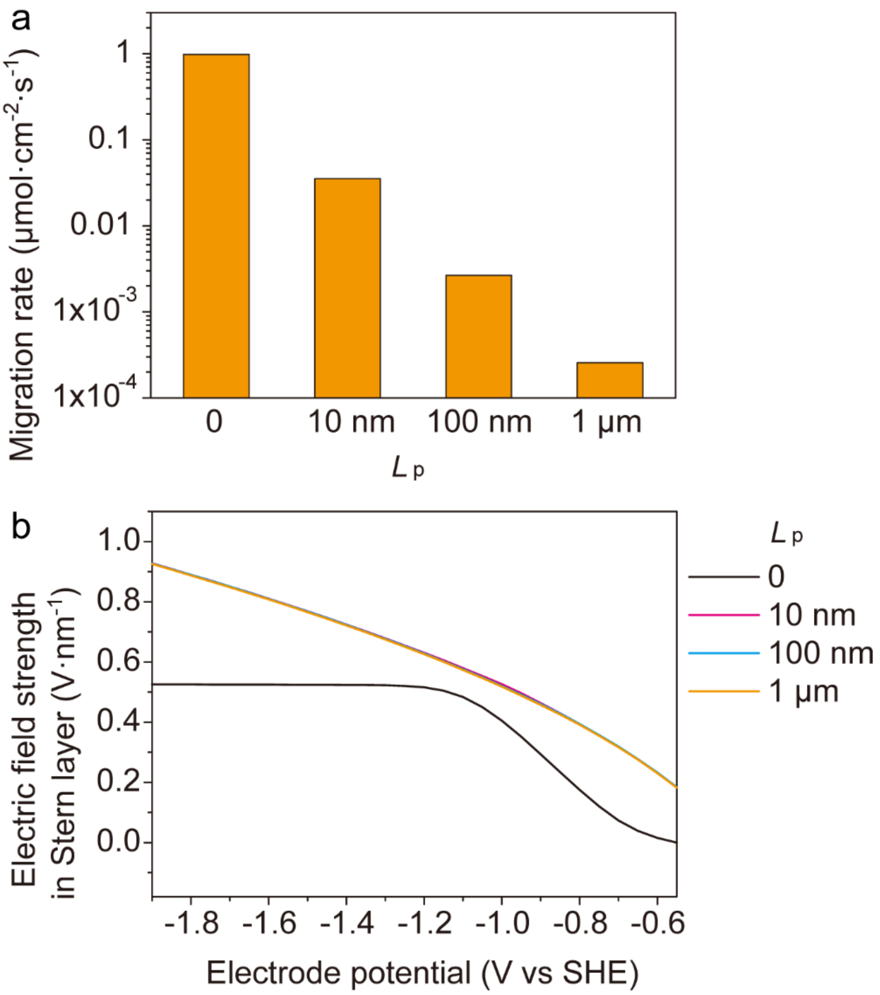

Figure S24. Simulated effects of the thickness of polymer layer (L_p) on the properties of Ag electrode in 10 mM HOTf. (a) The migration rate of H^+ at 2 μm from the OHP. (b) Plots of the electric field strength in Stern layer based on the electrode potential. ρ_p = +300 C·cm^{-3} for all the simulations.

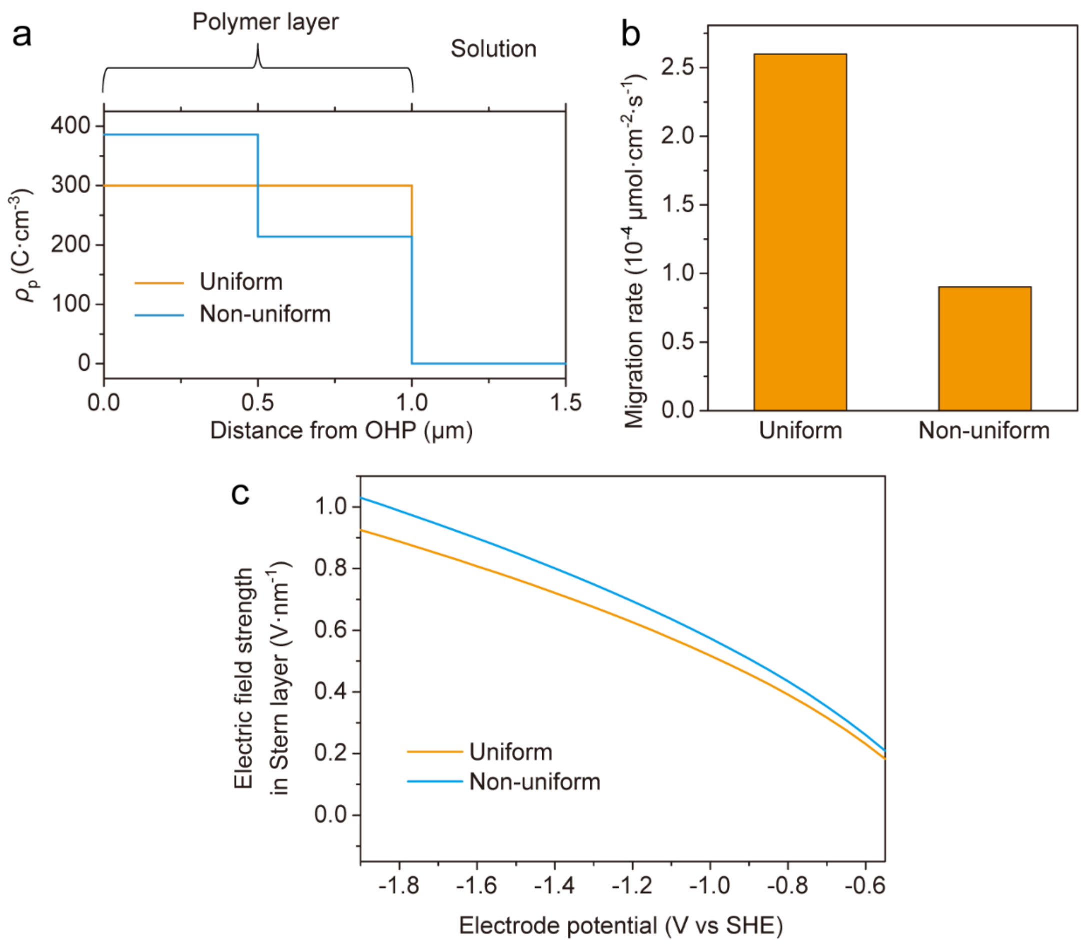

Figure S25. Simulated effects of the uniformity of the polymer layer on the properties of Ag electrode in 10 mM HOTf. (a) The proposed profiles of ρ_p for polymer with uniform charge density and non-uniform charge density. (b) The migration rate of H^+ at 2 μm from the OHP. (c) Plots of the electric field strength in Stern layer based on the electrode potential.

Table S1. Measured resistance of different electrolytes.

Electrolyte	Resistance (Ω)
0.1 M H_2SO_4	4.2 ± 0.2
1 M H_2SO_4	1.8 ± 0.1
0.1 M H_2SO_4 + 0.04 M K_2SO_4	4.0 ± 0.3
0.1 M H_2SO_4 + 0.1 M K_2SO_4	3.8 ± 0.2
0.1 M H_2SO_4 + 0.4 M K_2SO_4	3.3 ± 0.3
0.1 M $KHCO_3$	23 ± 2
0.1 M KOH	9.8 ± 0.6

Table S2. Parameters and coefficients used in the GMPNP simulations.

Parameter	Explanation	Value	Unit	Reference
D_{H+}	Diffusion coefficient of H^+ in water	9.311×10^{-9}	$m^2\cdot s^{-1}$	7
D_{K+}	Diffusion coefficient of K^+ in water	1.957×10^{-9}	$m^2\cdot s^{-1}$	7
D_{OH-}	Diffusion coefficient of OH^- in water	5.273×10^{-9}	$m^2\cdot s^{-1}$	7
D_{OTf-}	Diffusion coefficient of OTf^- in water	0.863×10^{-9}	$m^2\cdot s^{-1}$	10
z_{H+}	Number of charges carried by H^+	+1	1	
z_{K+}	Number of charges carried by K^+	+1	1	
z_{OH-}	Number of charges carried by OH^-	−1	1	
z_{OTf-}	Number of charges carried by OTf^-	−1	1	
a_{H+}	Effective solvated size of H^+	5.6×10^{-10}	m	7
a_{K+}	Effective solvated size of K^+	6.62×10^{-10}	m	7
a_{OH-}	Effective solvated size of OH^-	6×10^{-10}	m	7
a_{OTf-}	Effective solvated size of OTf^-	6×10^{-10}	m	10
V_p	Volume fraction of polymer	0.36	1	
L_p	Thickness of polymer layer	1×10^{-6}	m	
L_{tot}	Total length for simulation	9.8×10^{-6}	m	

Table S2 (continued). Parameters and coefficients used in the GMPNP simulations.

Parameter	Explanation	Value	SI unit	Reference
F	Faraday's constant	96485	$C \cdot mol^{-1}$	
R	Gas constant	8.314	$J \cdot mol^{-1} \cdot K^{-1}$	
T	Temperature	298.15	K	
N_A	Avogadro's number	6.0221×10^{23}	mol^{-1}	
k_{w1}	Rate constant of water dissociation	0.0204	$mol \cdot m^{-3} \cdot s^{-1}$	7
k_{w2}	Rate constant of neutralization	2.4×10^{6}	$mol^{-1} \cdot m^{3} \cdot s^{-1}$	7
ε_0	Permittivity of vacuum	8.8542×10^{-12}	$F \cdot m^{-1}$	7
ε_r	Relative permittivity of water	80.1	1	7
d	Thickness of Stern layer	4×10^{-10}	m	7
A	Pre-factor in Equation S12	5×10^{-9}	$m \cdot s^{-1}$	4
β	Electron transfer coefficient	0.42	1	4

Table S3. Simulated migration rate of H^+ (J_{Mig}) and the electric field strength in Stern layer (E_{Stern}) at -1.8 V vs SHE on Ag electrode covered by polymer layer with different charge density (ρ_p). The ratio between diffusion coefficients in polymer layer and in solution ($D_{i,p}/D_{i,s}$) was set to 0.1 or 0.5 and the simulation results are compared.

	$D_{i,p}/D_{i,s} = 0.1$		$D_{i,p}/D_{i,s} = 0.5$	
ρ_p (C·cm^{-3})	J_{Mig} (μmol·cm^{-2}·s^{-1})	E_{Stern} (V·nm^{-1})	J_{Mig} (μmol·cm^{-2}·s^{-1})	E_{Stern} (V·nm^{-1})
-100	1.10	0.343	1.10	0.343
0	0.56	0.387	0.91	0.407
+100	1.96×10^{-3}	0.550	9.64×10^{-3}	0.551
+300	2.60×10^{-4}	0.888	1.30×10^{-3}	0.888

Table S4. Simulated migration rate of H^+ (J_{Mig}) and the electric field strength in Stern layer (E_{Stern}) at -1.8 V vs SHE on Ag electrode covered by polymer layer with different charge density (ρ_p). The relative permittivity of the polymer layer ($\varepsilon_{r,p}$) was set to 80.1 or 50.0 and the simulation results are compared.

	$\varepsilon_{r,p}$ = 80.1		$\varepsilon_{r,p}$ = 50.0	
ρ_p ($C\cdot cm^{-3}$)	J_{Mig} ($\mu mol\cdot cm^{-2}\cdot s^{-1}$)	E_{Stern} ($V\cdot nm^{-1}$)	J_{Mig} ($\mu mol\cdot cm^{-2}\cdot s^{-1}$)	E_{Stern} ($V\cdot nm^{-1}$)
-100	1.10	0.343	1.10	0.404
0	0.56	0.387	0.55	0.446
+100	1.96×10^{-3}	0.550	1.87×10^{-3}	0.676
+300	2.60×10^{-4}	0.888	2.48×10^{-4}	1.070

Table S5. Simulated migration rate of H^+ (J_{Mig}) and the electric field strength in Stern layer (E_{Stern}) at -1.8 V vs SHE on Ag electrode covered by polymer layer with different charge density (ρ_p). The relative permittivity in Stern layer ($\varepsilon_{r,Stern}$) was set to 80.1 or 8.01 and the simulation results are compared.

	$\varepsilon_{r,Stern}$ = 80.1		$\varepsilon_{r,Stern}$ = 8.01	
ρ_p ($C \cdot cm^{-3}$)	J_{Mig} ($\mu mol \cdot cm^{-2} \cdot s^{-1}$)	E_{Stern} ($V \cdot nm^{-1}$)	J_{Mig} ($\mu mol \cdot cm^{-2} \cdot s^{-1}$)	E_{Stern} ($V \cdot nm^{-1}$)
-100	1.10	0.343	1.07	2.012
0	0.56	0.387	0.50	2.051
+100	1.96×10^{-3}	0.550	1.95×10^{-3}	2.240
+300	2.60×10^{-4}	0.888	2.60×10^{-4}	2.548

Supplementary References

1 Bard, A. J. & Faulkner, L. R. *Electrochemical methods: fundamentals and applications*. 2nd. edn, (John Wiley & Sons, Inc., 2001).

2 Xu, Y. *et al.* Self-Cleaning CO_2 Reduction Systems: Unsteady Electrochemical Forcing Enables Stability. *ACS Energy Letters* **6**, 809-815, (2021).

3 Wang, H., Thiele, A. & Pilon, L. Simulations of Cyclic Voltammetry for Electric Double Layers in Asymmetric Electrolytes: A Generalized Modified Poisson–Nernst–Planck Model. *The Journal of Physical Chemistry C* **117**, 18286-18297, (2013).

4 Qin, H.-G. *et al.* Quantitative Understanding of Cation Effects on the Electrochemical Reduction of CO_2 and H^+ in Acidic Solution. *ACS Catalysis* **13**, 916-926, (2023).

5 Bode, D. D., Andersen, T. N. & Eyring, H. Anion and pH effects on the potentials of zero charge of gold and silver electrodes. *The Journal of Physical Chemistry* **71**, 792-797, (1967).

6 Zhu, X., Huang, J. & Eikerling, M. Electrochemical CO_2 Reduction at Silver from a Local Perspective. *ACS Catalysis* **11**, 14521-14532, (2021).

7 Bohra, D., Chaudhry, J. H., Burdyny, T., Pidko, E. A. & Smith, W. A. Modeling the electrical double layer to understand the reaction environment in a CO_2 electrocatalytic system. *Energy & Environmental Science* **12**, 3380-3389, (2019).

8 Butt, E. N., Padding, J. T. & Hartkamp, R. Size-modified Poisson–Nernst–Planck approach for modeling a local electrode environment in CO_2 electrolysis. *Sustainable Energy & Fuels* **7**, 144-154, (2023).

9 Song, F. *et al.* An Unconventional Iron Nickel Catalyst for the Oxygen Evolution Reaction. *ACS Central Science* **5**, 558-568, (2019).

10 Vanýsek, P. Ionic conductivity and diffusion at infinite dilution, in *Handbook of Chemistry and Physics* (CRC Press, 1992).
